# The Issue of Monocyte Activation in ASD: Troubles with Translation

**DOI:** 10.33696/immunology.4.146

**Published:** 2022

**Authors:** R.J. Moreno, P. Ashwood

**Affiliations:** 1 Department of Medical Microbiology and Immunology, UC Davis, CA, USA; 2 M.I.N.D. Institute, University of California at Davis, CA, USA

**Keywords:** Autism spectrum disorder, Monocytes, Translation, Inflammatory bowel disease, Regulation

## Abstract

Autism spectrum disorder (ASD) prevalence has increased year on year for the past two decades and currently affects 1 in 44 individuals in the US. An increasing number of studies have pointed to increased immune activation as both an etiological agent and also involved in the ongoing pathological process of ASD. Both adaptive and innate immune responses have been implicated. Evidence of innate dysregulation has so far included increased production of innate inflammatory cytokines, increased cell numbers, and altered activation in monocytes in the blood and microglia in the brain. Suggesting an orchestrated innate immune response may be involved in ASD. Hughes et al. (2022) recently assessed transcriptome differences that could underlie altered activation of monocytes using next-generation bulk-RNA sequencing on isolated CD14+ monocytes at baseline and after activation with different Toll-like receptor agonists. Circulating CD14+ monocyte from children with autistic disorder (AD) and children diagnosed with perverse developmental disorder not otherwise specified (PDD-NOS) were found to differ in a number of activation pathways after gene enrichment analysis compared to typically developing children. There was an overall upregulation in translational machinery in both neurodevelopmental disorder groups, whereas typically developing children were downregulated, indicating an issue with monocyte activation. Several identified differentially expressed genes in monocytes were also identified as ASD at-risk genes, according to the Simons Foundation Autism Research Initiative (SFARI), and genes involved in inflammatory bowel diseases. This work implicates altered monocyte activation with a lack of regulation as a potential mechanistic issue in ASD. Future work is warranted to evaluate how monocyte regulatory mechanisms differ in ASD individuals.

## Commentary

Autism spectrum disorder (ASD) is diagnosed when communicative and social deficits, as well as restrictive and repetitive behaviors, present themselves during early childhood [1]. The Diagnostic and Statistical Manual V (DSM-V) diagnostic criteria for ASD is based on these behavioral symptoms, combining previous DSM-IV diagnosis with similar behavioral symptoms, like Pervasive Developmental Disorder, not otherwise specified (PDD-NOS) [2]. The heterogeneity of ASD is likely due to the summation of past definitions and complex genetic and environmental factors. ASD caused solely by single-genetic mutations account for approximately 5-10% of diagnoses, suggesting that there are significant environmental variables that contribute to its etiology [3]. Genes implicated in ASD etiology are also involved in the immune system, particularly in pathways that regulate immune activation. Several environmental factors related to ASD etiology also impact immunological activity/function. For example, one such factor is fetal exposure to infections or toxins that induce maternal-immune activation (MIA) [4] and can lead to an inflammatory cascade that alters neural and microglia activation in the offspring as well as long-term fetal and adult immune responses [5]. In a non-human primate model, offspring exposed to MIA showed elevated innate inflammatory cytokines 1 and 4 years post-birth associated with ASD-like behaviors [6].

Within the last 20 years, the immune system has been implicated in the etiology and severity of ASD. Immune dysfunction in ASD has been identified at several stages of life, present as early as gestation and into adulthood, suggesting chronic, ongoing immune activation [7]. Immune dysfunction is associated with worse ASD behaviors. Studies have documented an association between increased inflammatory cytokines like interleukin (IL)-1β and IL-6 with stereotyped behaviors [8]. In a prior study, male children with ASD could be endophenotyped as either having less or more severe behavioral impairments based on inflammatory cytokine production following lipopolysaccharide (LPS) stimulation of peripheral blood mononuclear cells (PBMC) [9]. Indeed, evidence suggests many of the cytokines implicated in ASD behaviors are produced by cellular components of the innate immune system, including blood monocytes [10].

Monocytes are innate myeloid cells that initiate immune responses. Monocytes possess pattern recognition receptors (PRRs) that recognize common structural carbohydrate, protein, or sugar moieties in microbes [11]. Toll-like receptors (TLRs) are a class of PRRs on the membrane and endosomes of monocytes that bind to various bacterial and viral components. TLR binding to pathogen-associated molecular patterns (PAMPs) initiates a signaling cascade that activates NF-κB, producing inflammatory cytokines like IL-1β, IL-6, and tumor necrosis factor-alpha (TNFα) [11]. In addition to producing cytokines, monocytes can differentiate into antigen-presenting macrophages and dendritic cells [12]. Monocyte-derived macrophages can enter tissues and coordinate local immune responses, whereas dendritic cells primarily communicate with the adaptive immune system. The dysregulation of monocytes, therefore, can have several implications for health and development. Several studies have identified various alterations in monocytes in ASD, including increased monocyte numbers, differential cytokine responses to TLR agonists, and increased IL-6 secretion after LPS stimulation [13–16]. In post-mortem brain tissue, increased expression of innate inflammatory genes and cytokines further implicate myeloid cell dysfunction in ASD pathology [17,18].

Recently, Hughes et al. applied next-generation sequencing technology to understand monocyte dysfunction at the transcriptome level under resting conditions and following activation. Children were recruited from typically developing (TD) children, children with autistic disorder (AD) or PDD-NOS children as part of the Autism Phenome Project [19]. CD14+ monocytes were isolated from the peripheral blood of AD, PDD-NOS, and TD individuals using positive selection to increase the purity of the cells studied. Monocytes were then either left unstimulated or activated with lipoteichoic acid (LTA), a TLR-2 agonist, or lipopolysaccharide (LPS), a TLR-4 agonist, for 24 hours. After incubation, the monocyte transcriptome was processed using 3′ bulk-RNA sequencing and analyzed for differential gene expression.

Gene expression in monocytes across the study groups did not differ significantly under basal (non-stimulated) resting conditions. Under inflammatory conditions, a number of differentially expressed genes (DEGs) involved in monocyte activation and translation differed between TD, AD, and PDD-NOS groups. Differences were evident based on whether TLR4 or TLR2 agonists were used for stimulation. In AD monocytes stimulated with theTLR4 agonist, 78 increased DEGs were observed and these enriched for the ‘pathogenic *E. coli* infection’, ‘innate immune response-activating signal transduction’, and ‘Fc receptor signaling pathway’ pathways. However, similar inflammatory pathways were not observed in the PDD-NOS group, suggesting key differences in inflammatory processes between AD and PDD-NOS. The 34 DEGs increased in PDD-NOS were enriched for the ‘ciliary base’ pathway. Genes previously associated with monocyte dysfunction in ASD were also upregulated, including the FAS cell surface death receptor (FAS), nuclear factor kappa B (NF-κB1), and TGF beta Kinase 3 (TAB3). In contrast to the AD and PDD-NOS groups, the 112 DEGs increased in TD monocytes stimulated with TLR4 agonist enriched for ‘supramolecular fiber and polymer’ pathways. As for decreased DEGs after TLR4 stimulation, pathway analyses in the PDD-NOS study group revealed that the ‘retinol metabolism’ pathway was significantly downregulated.

Stimulation with TLR2 agonists resulted in 94 increased DEGs that were seen in the AD group, but not associated with any identified pathways. In TD monocytes, 128 increased DEGs were increased after TLR2 stimulation and were enriched for the ‘cytokine receptor interactions’ pathway. Interestingly, the decreased DEGS from TD monocytes, regardless of activatory condition, were enriched in ‘translation regulation’ processes. Conversely, translational pathways dysregulated in AD and PDD-NOS monocytes included ‘rRNA processing’, ‘rRNA metabolic processes’, and ‘ribonucleoprotein complex biogenesis’ are increased. Altered translation suggests that while the activation may not significantly differ between groups, the ability of their monocytes to regulate the duration of their activation is impacted in AD compared with TD. While these data support the hypothesis of an altered activation state in monocytes, explanations for why this occurs need further investigation.

This study also compared CD14+ monocyte differential gene expression to published gene lists for DEGs in the brains of individuals with psychiatric and neurodevelopmental disorders, and ASD risk genes published by the Simons Foundation. In the AD group, several DEGs overlapped with known ASD or intellectual disability (ID) risk genes. Furthermore, an enrichment of DEGs in AD monocytes were associated with inflammatory bowel conditions. Gastrointestinal (GI) issues are more prevalent in the ASD population, therefore future studies evaluating the role of monocytes in GI dysfunction would be of interest [20].

One theory that may explain dysregulated monocyte activity pertains to trained immunity. Trained immunity is characterized as either innate tolerance or memory in cells of the innate immune system and is defined as cellular adaptions after the primary exposure to a stimulus that enables a faster secondary response [21]. Adaptations include the epigenetic modifications of genes coordinating immune function, protein translation, and cellular metabolism. In innate tolerance, the outcome of adaption results in reduced production of inflammatory cytokines and increased regulatory cytokines. Conversely, innate memory results in a more robust and faster inflammatory response after re-exposure. Given that monocytes from children with ASD are over-activated, the balance of monocyte tolerance and memory may be shifted away from tolerance and towards memory [15]. How innate memory is generated has not been extensively studied. However, in ASD, reports suggest low-level LPS-mediated endotoxemia and increased peripheral inflammatory cytokines are present that could combine to provide the conditions necessary to establish memory [8,22]. In addition, the relatively short lifespan of monocytes in circulation may mean that trained immunity may be occurring centrally during myeloid cell development. Central memory establishment may have implications for other myeloid cells, such as microglia. To determine if trained immunity is disrupted in ASD monocytes, epigenetic and metabolic studies in monocytes will be required.

Other mechanisms besides trained immunity may result in a stronger or prolonged activation. At the surface level, TLR surface density may influence activation outcomes. In schizophrenia, increased TLR-3 and TLR-4 expression are observed on unstimulated monocytes and associated with an early age of onset [23]. Interestingly, treatment with antipsychotics subsequently normalizes TLR expression [24]. TLR expression is also correlated with autoimmune pathology. In Type 1 diabetes, increased TLR-2 and TLR-4 expression was found in T1D patients and associated with increased NF-κB expression and IL-1β release [25]. Trained immunity may be playing a role in increased TLR signaling in disorders such as schizophrenia and TID, as it was previously shown to in Bacillus Calmette-Guèrin (BCG) vaccination study [26]. If altered TLR signaling is also observed in ASD, which may be likely given the evidence in Hughes et al. [19] and prior studies, innate memory may prove to be a contributor to myeloid cell dysfunction. Studies characterizing the surface phenotype of monocytes are still required to pursue this hypothesis.

Immune system abnormalities have been described in ASD, with a significant body of work describing the multiple immune system components impacted. However, few studies in the ASD literature have provided an in-depth evaluation of each immune component using unbiased methods. Hughes et al. [19] established that an aspect of monocyte dysfunction in ASD may be due to increased activation, loss of translation and loss of regulation. Dysregulated monocyte activity impacts their differentiation and how they interact with other components of the immune system, including the adaptive immune system disrupted in ASD. Future work will be required to identify how monocyte regulation is disrupted at the receptor, signaling and DNA level in neurodevelopmental disorders like ASD.
